# A Rare Presentation of Herpes Simplex Virus Encephalitis With Ischemic Stroke and Associated Acyclovir-Induced Acute Kidney Injury

**DOI:** 10.7759/cureus.71978

**Published:** 2024-10-20

**Authors:** Hamid Ullah, Zeeshan Ikhtiar, Hina Ikhtiar

**Affiliations:** 1 Internal Medicine, Russells Hall Hospital, The Dudley Group NHS Foundation Trust, Dudley, GBR; 2 Medicine, Lady Reading Hospital Medical Teaching Institution (MTI), Peshawar, PAK; 3 Biochemistry, Kabir Medical College, Gandhara University, Peshawar, PAK

**Keywords:** acyclovir in aki, drug-induced aki, hsv-1 encephalitis, hsv encephalitis, ischemic stroke

## Abstract

This case report describes a 26-year-old female presenting with clinical features of encephalitis, which progressed to ischemic stroke. In addition, she was started on intravenous acyclovir 750 mg three times a day (TID, 10 mg/kg body weight every eight hours, recommended dose), which led to the development of an acute kidney injury (AKI). Subsequently, conservative management and a reduction in the dosage of acyclovir, followed by stopping it, led to the recovery of the patient's renal function. Three days after completely stopping the drug administration, she was continued on a reduced dose of intravenous acyclovir, completing a 10-day course, with improvement in overall condition with some residual right upper limb and lower limb weakness and slurring of speech. This case signifies the importance of presentation, timely management, and prevention of acyclovir-induced AKI; in addition, it also reports a less common complication of herpes simplex virus encephalitis (HSVE), which is ischemic stroke. The management of all these herpes simplex virus (HSV-1)-related complications in a single case will add much to the existing literature.

## Introduction

Herpes simplex virus encephalitis (HSVE) is a necrotizing infectious disease of the brain tissue that, in most cases, is caused by the herpes simplex virus (HSV-1) [1]. It may be preceded by a flu-like illness and present acutely with altered mental status, seizures, fever, and headache. Its pathological manifestations include perivascular inflammation and edema, generally having high rates of mortality and intensive care unit (ICU) admission. Studies highlighting HSV-related cerebrovascular disease have reported stroke, whether ischemic or hemorrhagic, as a rare and serious complication. An analysis of 4,800+ cases of HSVE in the USA reported an incidence of intracranial hemorrhage and ischemic stroke in 2.7% and 5.6%, respectively [2, 3].

Acyclovir is used as the drug of choice in HSVE and has a proven mortality benefit when therapy is started as soon as possible, preferably within 48-72 hours, even before the diagnosis has been confirmed [4,5]. Intravenous acyclovir should be started at 10 mg/kg body weight every eight hours of the recommended dose and given for at least 14 days to reduce the risk of relapse [6, 7]. Its primary route of excretion is through the kidneys; therefore, acute kidney injury (AKI) is a relatively common complication reported in 10%-48% of patients. [8]

We report a unique case of HSVE progressing to ischemic stroke (all ethical standards were met and patient consent was obtained) and complicated by acyclovir-induced AKI.

## Case presentation

A 26-year-old unmarried female with no previous comorbidity and normal menstrual history presented to the emergency department with a three-day history of severe headache, low-grade fever, and nausea with vomiting, followed by three episodes of generalized tonic-clonic (GTC) fits and loss of consciousness the night before her presentation. This was preceded by an episode of flu-like illness 11 days ago, which resolved spontaneously with symptomatic treatment.

On examination, the patient was febrile and lethargic but arousable with incomprehensible verbal sounds, and bilateral pupils which were reactive to light. She was vitally stable, with a Glasgow Coma Scale (GCS) of 9/15 and a positive neck stiffness. The patient had limitations of movement overall, more on the right side with the power of 1/5 in the upper and lower limbs; the right plantar was upgoing while the left was mute. Her initial laboratory investigations revealed a normal complete blood count, renal function tests, liver function tests, and metabolic profile (blood sugar, serum electrolytes, including calcium and magnesium). She had a normal chest X-ray and electrocardiogram (ECG). Her findings were suggestive of stroke; therefore, a CT scan of the brain without contrast was advised which reported findings of bilateral frontal and left parietal hypodense areas. Neurology colleagues and a radiologist labeled her a case of left middle cerebral artery (MCA) infarct with non-specific left parietal lobe ischemia.

**Figure 1:**
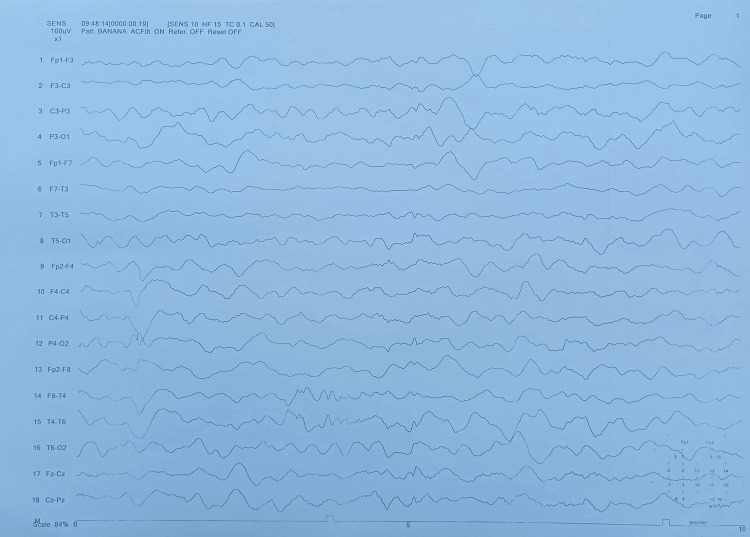
The EEG shows an encephalopathic pattern Slow and abnormal background with generalized diffuse delta range activity recorded

**Figure 2:**
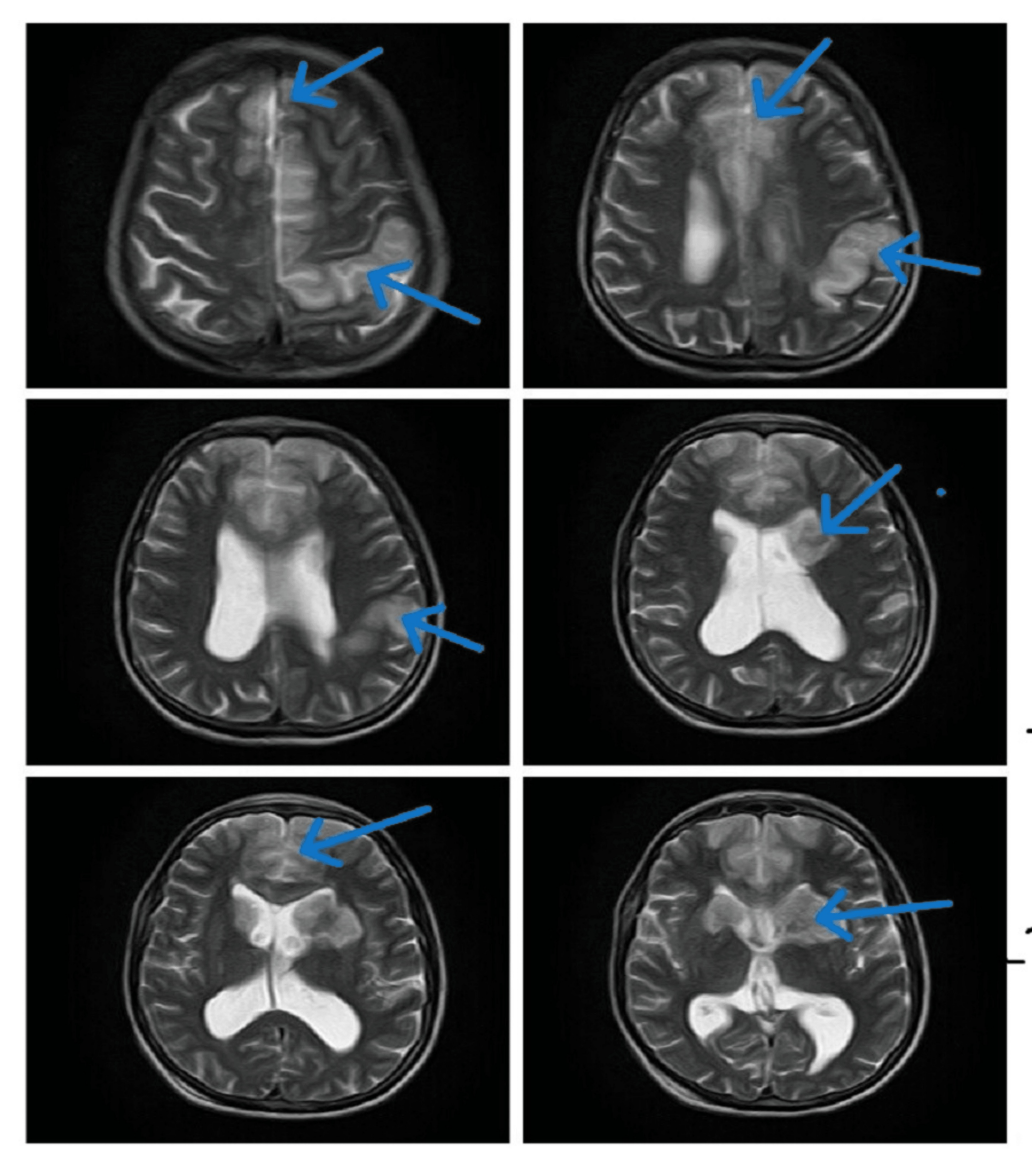
T2-weighted imaging (T2WI) MRI (axial view) shows multifocal ischemia (blue arrows)

**Figure 3:**
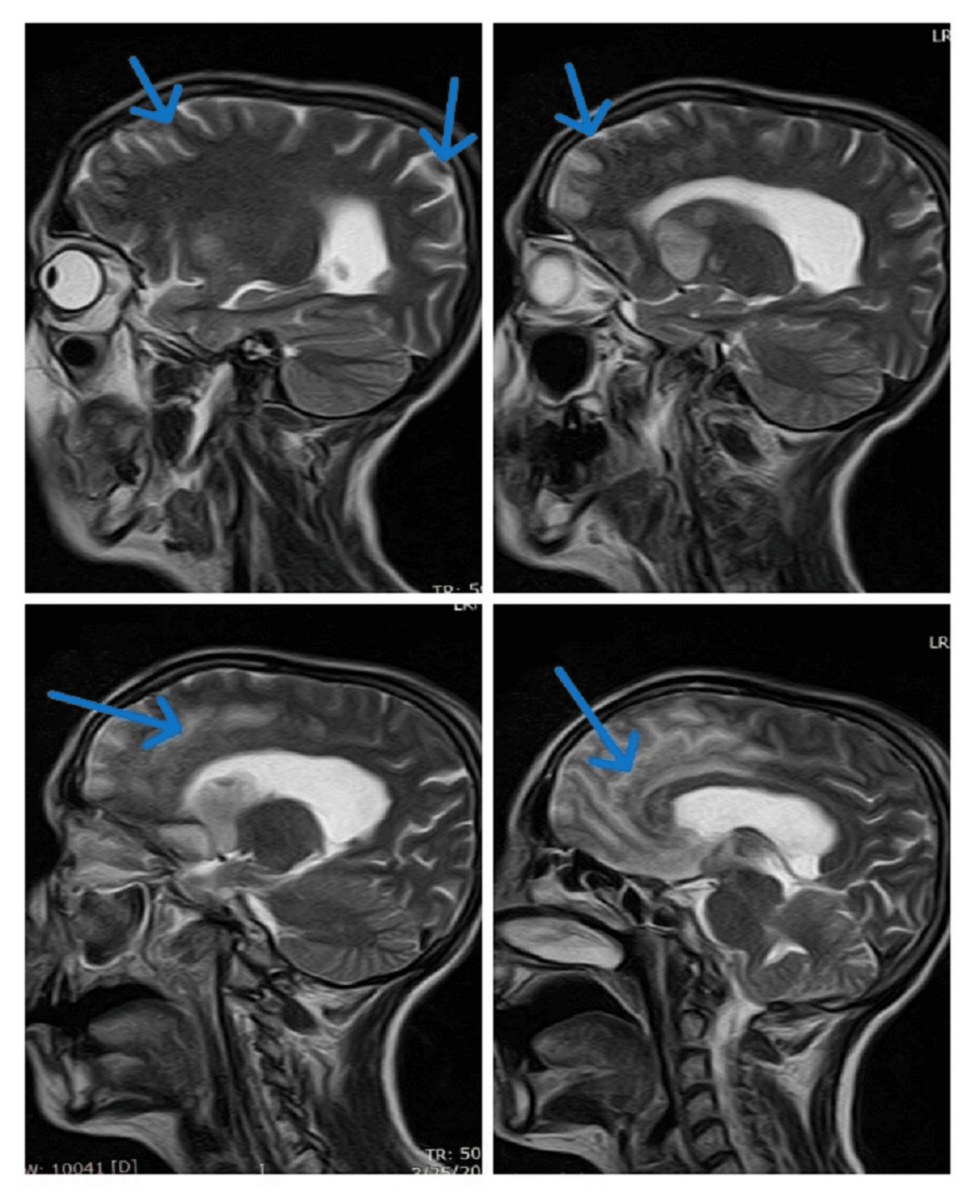
T2-weighted imaging (T2WI) MRI (sagittal view) shows multifocal ischemia (blue arrows)

**Figure 4:**
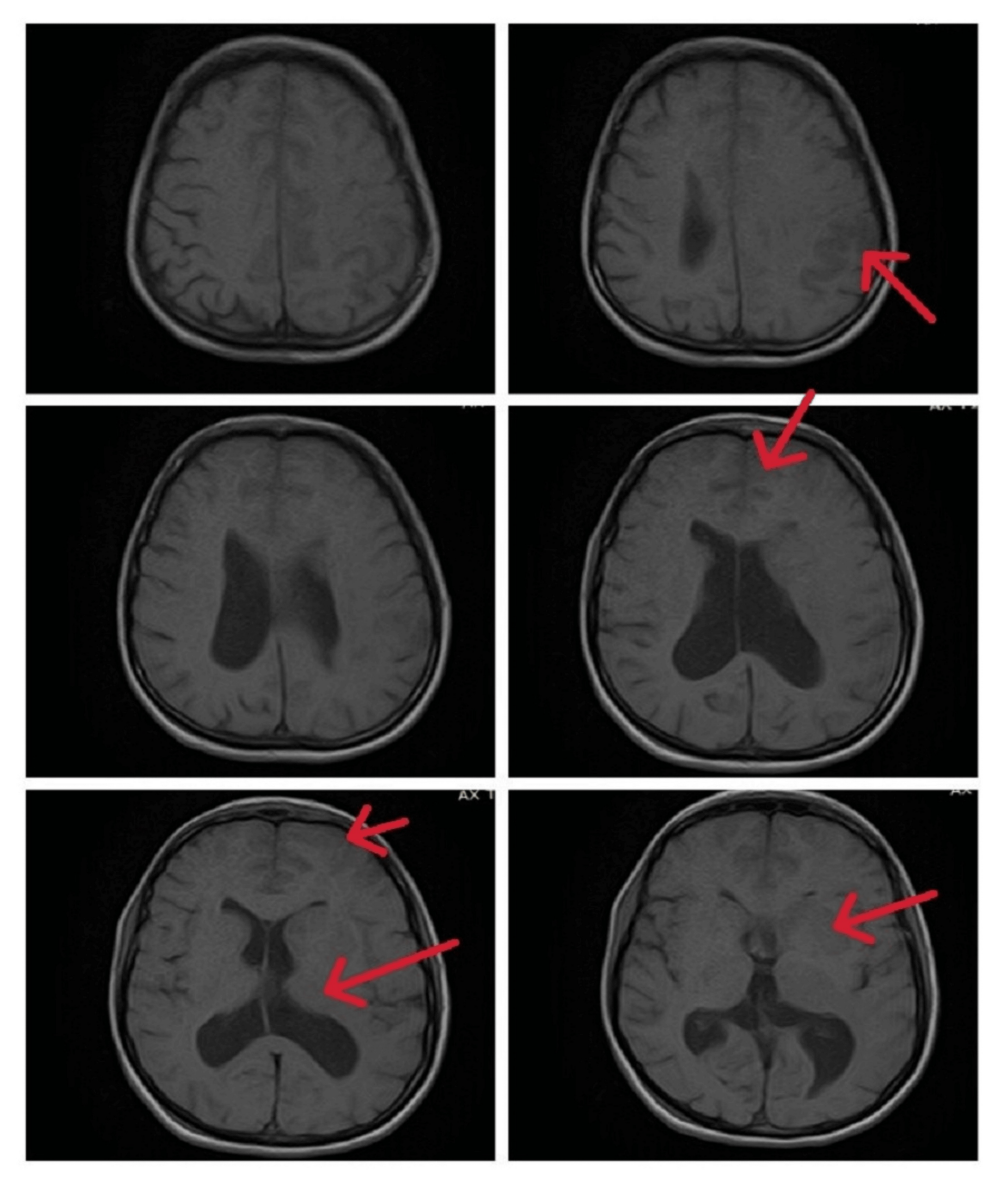
T1-weighted imaging (T1WI) MRI shows areas of hypointensities and multifocal ischemia (red arrows)

**Figure 5:**
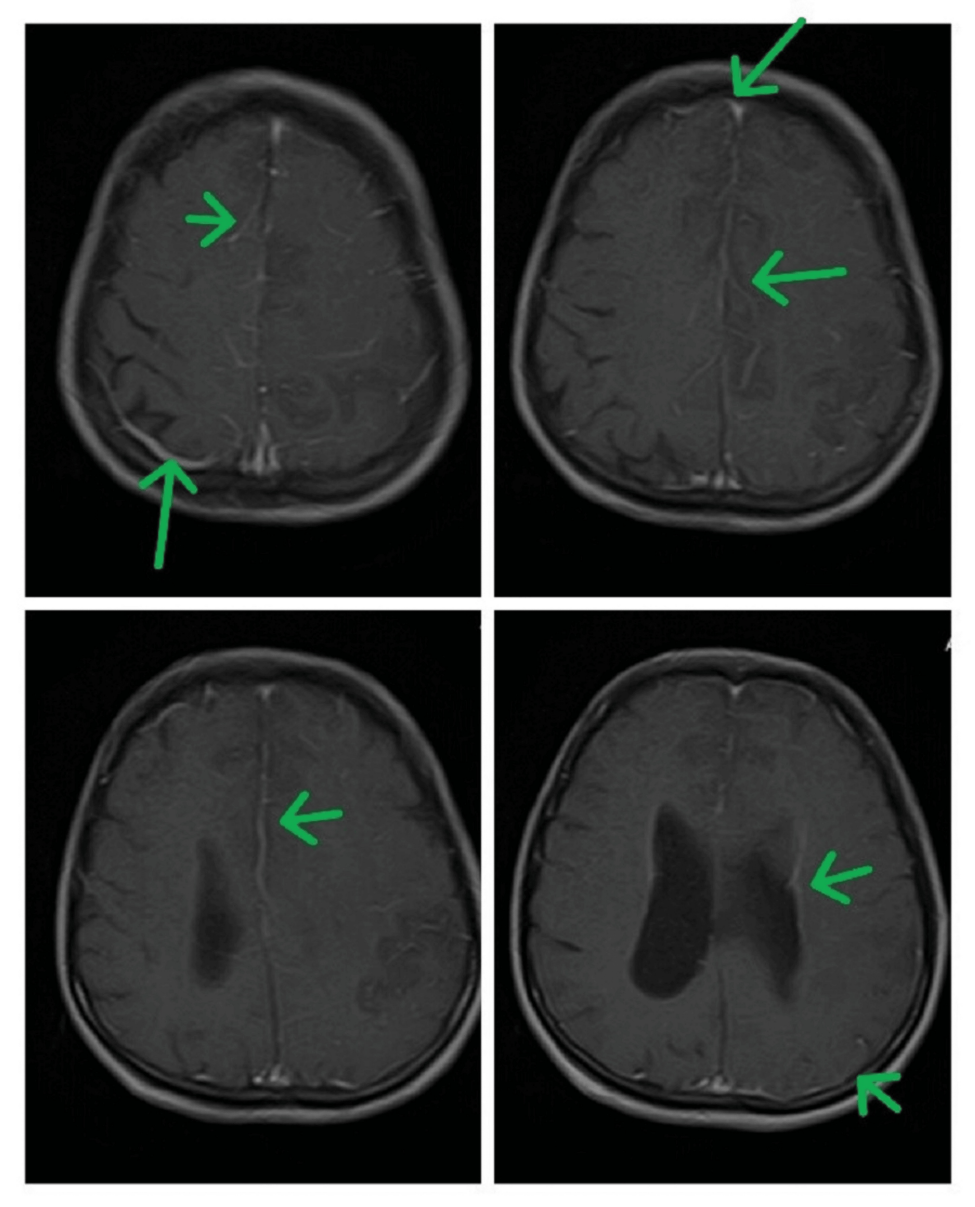
T1-weighted imaging (T1WI) MRI post-contrast images show leptomeningeal enhancement (green arrows)

We made a provisional diagnosis of ischemic stroke/meningoencephalitis, and her lumbar puncture and a brain MRI with contrast were performed. She again had a single episode of GTC fit (terminated with 10 mg IV diazepam stat); therefore, an electroencephalogram (EEG) was also planned. She was started on prophylactic IV antibiotics (cefotaxime 2 mg four times a day (QID), 8 mg daily, divided into four doses recommended by British National Formulary (BNF)/National Institute for Health and Care Excellence (NICE)), antiplatelet therapy (IV valproic acid 250 mg two times a day (BID), IV anti-epileptics, and IV acyclovir 750 mg three times a day (TID, 10 mg/kg body weight every eight hours, recommended dose, weight 72 kg, dose 720 mg) [6]. In the meantime, her echocardiography and ultrasound Doppler carotids were done and reported as normal. Her EEG showed an encephalitic pattern (Figure 1), and cerebrospinal fluid (CSF) routine examination (r/e) revealed a raised cell count of 37/mm3, predominantly lymphocytes, normal glucose, and mildly raised protein, suggesting viral encephalitis or meningitis. Her CSF polymerase chain reaction (PCR) for HSV-1 report returned positive two days later, and her brain MRI with contrast reported findings suggestive of meningoencephalitis with multifocal ischemic changes (Figures 2-5). Her IV antibiotics were stopped, and she was continued on IV acyclovir 750 mg TID (10 mg/kg body weight every eight hours) at the recommended dose.

The next day (48 hours after initiation of IV acyclovir), the patient's urine output decreased to 300 ml/24 hours, and her serum creatinine rose to 2.9 mg/dl (a total dose of 3,750 mg). The nephrology unit was consulted, and after consensus, the patient was started on IV fluids and diuretic therapy, and her IV acyclovir dose was decreased to 500 mg TID. Her urine r/e was reported to be normal with no casts or crystals. The next day, her serum creatinine was reported at 3.8 mg/dl, prompting us to withhold her IV acyclovir (total dose amounting to 4,750 mg). IV fluids and diuretics were continued. Over the course of the next three days, her creatinine decreased to 1.7 mg/dl, and her urine output increased to 2,950 ml/24 hours. She was again started on 500 mg of IV TID acyclovir while maintaining IV fluids with strict monitoring of renal function and urine output. She completed a 10-day course of IV acyclovir in total and showed marked improvement in her level of consciousness. After a total of 16 days of hospital stay, on discharge, her renal functions were normal with a serum creatinine of 0.9 mg/dl, and there was some residual right upper and lower limb weakness, slurring of speech, and cognitive impairment. She was called for a two-month follow-up.

## Discussion

Herpes simplex virus encephalitis is a necrotizing infectious disease of the brain tissue, which in most cases is caused by HSV-1. It may be a primary infection or a reactivation of a dormant virus and is clinically defined by a preceding flu-like illness, altered mental status, focal or generalized seizures, fever, and headache that develop acutely. Cerebrovascular complications in the form of ischemic and hemorrhagic strokes are common with both HSV-1 and HSV-2 [1]. Among these, more than 80% of reported complications with HSV-1 are hemorrhagic, while HSV-2 is the most prevalent agent in the ischemic manifestations (70%) [2, 9, 10-14]. Infarction is frequently multifocal, mostly related to cerebral large vessel vasculitis [2]. However, our case report showed a contrasting presentation of multifocal ischemia on the background of HSVE, a less common complication.

Intravenous acyclovir is the drug of choice for the treatment of HSVE. Its primary route of excretion is through glomerular filtration, and renal tubular secretion via the kidneys accounts for up to 90%; therefore, nephrotoxicity in patients treated with IV acyclovir is primarily caused by tubular deposition of acyclovir crystals or direct tubular toxicity. In our setup, polarizing light microscopy was unavailable, justifying the absence of crystals on urine analysis. In addition, IV therapy is necessary to achieve effective blood concentrations, which explains why crystal nephropathy is more common. This complication can be potentiated by dehydration, rapid intravenous infusion, pre-existing AKI/chronic kidney disease (CKD), and concurrent use of other nephrotoxic agents. Therefore, it is important to establish euvolemia before medication administration, infuse IV acyclovir slowly, monitor renal function in patients on acyclovir, and timely adjust the dose for renal function [5,15-17]. In our case report, prompt use of IV fluids and IV diuretics, adjustment of the acyclovir dose, and rigorous monitoring of the creatinine level and urine output resulted in a quick recovery of the patient’s renal function.

## Conclusions

Our case report signifies the importance of presentation, timely management, and prevention of acyclovir-induced AKI. Therefore, it is important to establish euvolemia before medication administration, infuse the drug slowly, make timely adjustments to the dose for renal function, and avoid other nephrotoxic agents. However, once AKI develops, maintaining urine output at a high rate with IV fluids and diuretics, along with cessation of acyclovir if needed, must be ensured. In addition, this case also highlights a less common complication of HSV-1 encephalitis in the form of ischemic stroke; therefore, there is a need to investigate and unravel the mechanisms of such complications.

## References

[1] Roçi E, Dodaj S, Vyshka G (2023). Herpes simplex virus encephalitis mimicking acute ischemic stroke. Surg Neurol Int.

[2] Hauer L, Pikija S, Schulte EC, Sztriha LK, Nardone R, Sellner J (2019). Cerebrovascular manifestations of herpes simplex virus infection of the central nervous system: a systematic review. J Neuroinflammation.

[3] Jouan Y, Grammatico-Guillon L, Espitalier F, Cazals X, François P, Guillon A (2015). Long-term outcome of severe herpes simplex encephalitis: a population-based observational study. Crit Care.

[4] Gnann JW Jr, Whitley RJ (2017). Herpes simplex encephalitis: an update. Curr Infect Dis Rep.

[5] Lee EJ, Jang HN, Cho HS, Bae E, Lee TW, Chang SH, Park DJ (2018). The incidence, risk factors, and clinical outcomes of acute kidney injury (staged using the RIFLE classification) associated with intravenous acyclovir administration. Ren Fail.

[6] Stahl JP, Mailles A, De Broucker T (2012). Herpes simplex encephalitis and management of acyclovir in encephalitis patients in France. Epidemiol Infect.

[7] Bell DJ, Suckling R, Rothburn MM (2009). Management of suspected herpes simplex virus encephalitis in adults in a U.K. teaching hospital. Clin Med (Lond).

[8] Richelsen RK, Jensen SB, Nielsen H (2018). Incidence and predictors of intravenous acyclovir-induced nephrotoxicity. Eur J Clin Microbiol Infect Dis.

[9] Zepper P, Wunderlich S, Förschler A, Nadas K, Hemmer B, Sellner J (2012). Pearls & oy-sters: cerebral HSV-2 vasculitis presenting as hemorrhagic stroke followed by multifocal ischemia. Neurology.

[10] Sivasankar C, White K, Ayodele M (2019). An unusual etiology of acute spontaneous intracerebral hemorrhage. Neurohospitalist.

[11] Sakaguchi J, Yonemura K, Hashimoto Y, Hirano T, Uchino M (2005). Herpes simplex encephalitis originating from bilateral thalamic lesions with hemorrhagic component (Article in Japanese). Rinsho Shinkeigaku.

[12] ElShimy G, Mariyam Joy C, Berlin F, Lashin W (2017). Intracranial hemorrhage complicating herpes simplex encephalitis on antiviral therapy: a case report and review of the literature. Case Rep Infect Dis.

[13] Sas AM, Niks EH, Lequin MH, Catsman-Berrevoets CE, de Wit MC (2009). Herpes simplex virus type-1 encephalitis and occipital ischemic stroke. Pediatr Neurol.

[14] Tsuboguchi S, Wakasugi T, Umeda Y, Umeda M, Oyake M, Fujita N (2017). Herpes simplex encephalitis presenting as stroke-like symptoms with atypical MRI findings and lacking cerebrospinal fluid pleocytosis (Article in Japanese). Rinsho Shinkeigaku.

[15] Al-Alawi AM, Al-Maqbali JS, Al-Adawi M, Al-Jabri A, Falhammar H (2022). Incidence, patterns, risk factors and clinical outcomes of intravenous acyclovir induced nephrotoxicity. Saudi Pharm J.

[16] Chávez-Iñiguez JS, Medina-Gonzalez R, Aguilar-Parra L, Torres-Vázquez EJ, Maggiani-Aguilera P, Cervantes-Pérez E, García-García G (2018). Oral acyclovir induced hypokalemia and acute tubular necrosis a case report. BMC Nephrol.

[17] Fleischer R, Johnson M (2010). Acyclovir nephrotoxicity: a case report highlighting the importance of prevention, detection, and treatment of acyclovir-induced nephropathy. Case Rep Med.

